# The incidence of construction site injuries to women in Delhi: capture-recapture study

**DOI:** 10.1186/s12889-021-10930-6

**Published:** 2021-05-03

**Authors:** Sajjan S. Yadav, Phil Edwards, John Porter

**Affiliations:** 1 Department of Expenditure, Ministry of Finance, Government of India, House No-1, Vinay Marg, Chanakya Puri, North Block, New Delhi, India; 2 Epidemiology and Statistics, Department of Population Health, London School of Hygiene and Tropical Medicine, Keppel Street, London, WC1E 7HT UK; 3 Department of Global Health and Development, London School of Hygiene and Tropical Medicine, Keppel Street, London, WC1E 7HT UK

**Keywords:** India, Construction, Injuries, Capture re-capture, First information reports, Safety

## Abstract

**Background:**

In India, the construction sector provides the main alternative to agricultural work - seasonal migration to and from construction work is widespread and construction work remains the second-largest employer of women in the country behind agriculture. Occupational injuries, which kill over 300,000 people annually, are a serious public health concern. However, data on construction site injuries to women are lacking, as India does not publish statistics on occupational injuries and illnesses. Our objectives were to: Estimate the number of women injured in construction site accidents in Delhi; and to estimate and compare the annual construction site injury rates per 100,000 workers of males and females in Delhi.

**Methods:**

We conducted a two-sample capture-recapture study using data for accidents reported to the Delhi Police, Employee State Insurance Corporation (ESIC), and Commissioners of Workmen Compensation (CWC) of Delhi Government. The capture-recapture method has been used in epidemiology, to estimate morbidity and mortality using multiple, overlapping, but incomplete data sources. This study is based on the injuries reported from construction site accidents in Delhi in 2017. We linked the data from each of the data sources using the name, gender, and age of each injured person, the date and place of the accident, and the name of the employer. We used the Chapman estimator to estimate the total incidence of construction injuries in Delhi.

**Results:**

We estimated that there was a total of 37 female construction site workers injured (17 fatal and 20 non-fatal) in Delhi in 2017. There was a total of 1043 male construction site workers injured (236 fatal and 807 non-fatal). FIRs ascertained two-thirds (68%) of all injuries to females but only one third (34%) of those to males. The annual construction site injury rate per 100,000 workers of females was 82.26 (95%CI: 57.92 to 113.39). The annual construction site injury rate per 100,000 workers of males was 146.5 (95%CI: 137.7 to 155.6). There was strong evidence (*p* = 0.001) that the overall construction site injury rate per 100,000 workers of females was about one half the rate of males [rate ratio 0.56 (95%CI: 0.40 to 0.78)]. There was no evidence (*p* = 0.601) that the rates of fatal injuries differed in males and females (rate ratio 1.14 (95%CI: 0.70 to 1.87).

**Conclusions:**

This study is the first to estimate the incidence of injuries to female construction site workers in India. The overall injury rate of female construction workers was over half as great as the rate of males. This implies that female construction workers face a not insignificant risk. Hence, safety measures (e.g., personal protective equipment) that are appropriate and culturally acceptable to Indian women are needed.

## Introduction

In India, the construction sector provides the main alternative to agricultural work - seasonal migration to and from construction work is widespread and construction work remains the second-largest employer of women in the country behind agriculture [1]. Women are often required to do the lift-and-carry work, climbing scaffoldings with bricks and soil on their heads [2].

Occupational injuries, which kill approximately 335,000 persons annually, are a serious public health concern [3]. With a 30–40% share, the construction industry is the lead contributor to occupational injuries [4, 5]. However, data on construction site injuries to women are lacking, as India does not publish statistics on occupational injuries and illnesses [5].

Delhi, the capital of India entices construction workers due to high wages and geographical advantage [6]. Previous studies have estimated that that 619,767 persons were employed in the construction sector in Delhi in the year 2012 [7]. It was estimated that between 2008 to 2012, 256 fatal injuries were reported annually at construction sites in Delhi [7]. However, there is a lack of studies on the prevalence of construction site injuries in Delhi, particularly studies of construction site injuries involving women.

Health records are a potential source of data on injuries across the globe. However, in India health records are generally maintained in paper format only. Even where hospital functioning has been computerised, records are often stored in disparate computer systems without inter-operability or cross-sharing [8]. The reports of accidents and injuries and compensation claims filed with various responsible authorities are a potential data source on injuries in India. Police records are another potential injuries data source because all First Information Reports (FIRs) registered by the police are required to be uploaded on a centralised, web-based, system – the Crime and Criminal Tracking Network & Systems (CCTNS), put in place by the Ministry of Home Affairs, Government of India [9].

Information relating to an accident, whether received orally or in writing, is required under the Indian Penal Code 1860 to be recorded by the officer in-charge of a police station, in a prescribed format, commonly known as the ‘First Information Report’ (FIR) [10]. Information on injuries can be reliably extracted from FIRs using a data extraction tool [11].

In this study we investigate the incidence of construction site injuries to women in Delhi. Specifically, we aimed:

- to estimate the number of women injured in construction site accidents in Delhi;
- to estimate and compare the annual construction site injury rates per 100,000 workers of males and females;
- to estimate the completeness of ascertainment by FIRs of injured female construction site workers.

## Methods

### Study design

This was a two-sample capture-recapture study. The Capture-recapture method has been used in epidemiology, to estimate morbidity and mortality using multiple, overlapping, but incomplete data sources [12]. The method has also been used to estimate injury morbidity and mortality [13–15].

#### Definitions

Building or other construction work was defined as the construction, alteration, repairs, maintenance or demolition of buildings, streets, roads, railways, tramways, airfields, irrigation, drainage, embankment and navigation works; flood control works; generation, transmission and distribution of power; water works; oil and gas installations; electric lines; wireless, radio, television, telephone, telegraph and overseas communications; dams, canals, reservoirs, watercourses; tunnels, bridges, viaducts, aqueducts, pipelines; towers, cooling towers, and transmission towers [12].

Building or construction worker was defined as a person who is employed to do any skilled, semi-skilled or unskilled work which could be manual, supervisory, technical or clerical in connection with any building or other construction [16]. The definition excluded persons employed mainly in a managerial or administrative capacity, visitors to construction sites, neighbours and passer-by.

Construction site was defined as a place where any building or other construction work is occurring [16].

Accident was defined as “an unintentional event characterized by the sudden release of an external force or impact, which can manifest itself as body injury” [17].

Injury was defined as “a bodily lesion resulting from acute overexposure to energy, which interacts with the body in amounts or rates that exceed the threshold of physiological tolerance” [17]. .Injuries included in this study did not include any psychological harms. Diseases or mental health conditions were also excluded.

### Data sources

We obtained the data for accidents reported to the police, Employee State Insurance Corporation (ESIC), and Commissioners of Workmen Compensation (CWC) of Delhi Government from 1st January 2017 to 31st December 2018. The study was restricted to accidents occurring between 1st January 2017 to 31st December, 2017. Accidents that happened during this period but were reported in the year 2018 to any of the three authorities were included. The accidents which happened before 1st January 2017 but reported during the year 2017 were excluded from the study. FIRs of construction site accidents were downloaded from the Delhi Police website and data were extracted [18].

The first sample comprised data on construction site injuries extracted from the FIRs. The second ‘recapture’ sample comprised data on construction injuries reported to the ESIC, combined with data on claims for compensation filed with the CWC. This combination of datasets, for the ‘recapture’ sample was made because ESIC largely covers workers employed in the ‘organised’ sector (i.e., enterprises employing 10 or more workers), while people going to the CWC with claims are largely from the ‘unorganised’ sector (i.e., enterprises employing fewer than 10 workers) [19]. Thus, once any duplicates had been removed, the combination of these two datasets provided a more complete, and independent source of data for this study. However, we did not find any duplicates in the ESIC and Workers’ compensation databases.

#### Missing records

Documents of 90 FIRs were found missing on Delhi Police website which were obtained from the police station concerned. Similarly, one incident report was found missing in case of ESIC which was obtained from the regional offices of ESIC.

### Record linkage

We created separate lists, using Microsoft Excel, for the data extracted from each of the two samples, described above. Each list contained the: Name, gender, and age of each injured person, the date and place of the accident, the name of the employer and the source of the data.

#### Matching

Record matching was achieved using a careful two-stage linkage process: In addition to the first author, a person employed for the extraction of data from the FIRs also independently conducted the record linkage. There were no differences in the results of the matching by the two people doing the matching.

##### Linkage stage 1

In the first stage of linkage, we generated matched pairs of records by matching on four identifying variables: (i) name, (ii) gender, (iii) age of the injured person, and (iv) date of the accident. Our aim was to produce a manageable number of possible matched pairs, without excluding any correct matches. While matching, we allowed for some disagreements in all variables, except gender, to allow for inaccuracies in the recording or for genuine differences between the two samples. Information on gender was available in all records. For (iv) date of the injury event, we allowed for differences of up to 3 days, as injuries may be reported later and the victims may not be able to recall the precise date of the accident. For (ii) age of the injured person, we allowed for differences of plus or minus 5 years, as age was not recorded in either of the two lists on the basis of date of birth, but instead by an estimate of age given by the injured person, or by friends or relatives of the injured person. Spelling errors in (i) name of the injured person were ignored and the name was considered as matched if it sounded phonetically the same in the two lists.

This linkage process resulted in some police records in the first sample matching more than one {ESIC + CWC} record in the second sample, and some {ESIC + CWC} records matched more than one police record.

##### Linkage stage 2

In the second stage of linkage, we resolved cases with more than one match using information from two additional variables: (v) Locality of the injury event and (vi) Name of employer; Spelling errors in these variables were ignored. When the name of the injured person was not available in either or both lists, an injured person was considered as matched if the other five variables matched.

#### Setting

This study was conducted in Delhi, India based on the records of building or construction workers injured at a building or other construction work sites in Delhi from 1st January to 31st December, 2017.

### Statistical methods

#### Estimation of total number of construction injuries in Delhi

We used the Chapman estimator to estimate the total number of construction injuries in Delhi [20, 21]. We quantified the precision of the estimate by calculating a confidence interval using a variance-based approach [22].

After estimating the total number of injuries, we calculated the percentage of all injuries captured by FIRs to estimate the completeness of ascertainment of injuries by FIRs.

#### Denominators for rates per 100,000 construction workers

Authoritative data on the size of the construction workforce in Delhi and its distribution by gender are not routinely available. Therefore, we estimated the population of construction workers in Delhi according to gender based on the total population of Delhi as per the 2011 population census, the labour force participation rate, and the proportion of workers in the labour force that works in construction. The population of Delhi as per the 2011 population census was 16.78 million [23]. The estimated annual growth rate of the population was 2.12% [23]. The labour force participation in Delhi was 59.7% for males and 12.4% for females [23]. In urban areas in India, the proportion of workers in the construction sector is 11.7% among males and 4.1% among females [24]. 97.5% of the population of Delhi lives in urban areas, so we assumed that the entirety of Delhi is an urban area [23]. Using the estimated population as denominators, we estimated the construction site injury rate per 100,000 workers in 2017.

## Results

Our analysis of FIR data found that 374 construction workers had suffered an injury in Delhi in 2017: 25 (7%) were female and 349 (93%) were male; 264 of these injuries were non-fatal and 110 were fatal (Table 1). Our analysis of the ESIC and CWC data found that 80 construction workers had suffered an injury in Delhi in 2017: 2 (3%) were female and 78 (97%) were male. No report of a non-fatal injury to a female construction worker was received either by the ESIC or the CWC. Record linkage of injured workers in the two samples produced 20 matches of fatal injuries and 9 matches of non-fatal injuries. There were no matches of female construction workers (Table 1).

**Table 1:** Construction workers injured in Delhi in 2017

Source	Number of workers injured								
	Fatal			Non-fatal			All injuries		
	Male	Female	Total	Male	Female	Total	Male	Female	Total
FIRs (% ascertainment)	105 (44.5%)	5 (29%)	110 (43%)	244 (30%)	20 (100%)	264 (30%)	349 (34%)	25 (68%)	374 (33%)
ESIC + CWC combined (% ascertainment)	46 (19.5%)	2 (11.8%)	48 (18.6%)	32 (4%)	0 (0%)	32 (3.7%)	78 (7%)	2 (5%)	80 (8%)
Matched records	20	0	20	9	0	9	29	0	29
Capture-recapture analysis (Chapman estimator) estimate of total numbers (95% CI)	**236** (188–340)	**17** (7–662)	**258** (204–373)	**807** (536–1522)	**20** (19–1102)	**873** (579–1648)	**1043** (731–1263)	**37** (32–2762)	**1131** (800–1390)

We estimated that there was a total of 37 female construction site workers injured (17 fatal and 20 non-fatal) in Delhi in 2017. There was a total of 1043 male construction site workers injured (236 fatal and 807 non-fatal). FIRs thus ascertained two-thirds (68%) of all injuries to females but only one third (34%) of those to males (Table 1).

The estimated numbers of construction workers and estimated injury rates in Delhi in 2017 are shown in Table 2.

**Table 2:** Estimated number of construction workers and injury rates in Delhi, 2017

	Male	Female	Rate Ratio (95%CI)
Construction workers in 2017	711,960	44,978	–
**Fatal**	236	17	–
Rate per 100,000; 95%CI	33.15 (29.05 to 37.66)	37.8 (22.02 to 60.52)	1.14 (0.70 to 1.87); p = 0.601
**Non-fatal**	807	20	–
Rate per 100,000; 95%CI	113.35 (105.66 to 121.45)	44.47 (27.16 to 68.67)	0.39 (0.25 to 0.61); *p* < 0.001
**All injuries**	1043	37	–
Rate per 100,000; 95%CI	146.5 (137.7 to 155.6)	82.26 (57.92 to 113.39)	0.56 (0.40 to 0.78); p = 0.001

The annual construction site injury rate per 100,000 workers of females was 82.26 (95%CI: 57.92 to 113.39). The annual construction site injury rate per 100,000 workers of males was 146.5 (95%CI: 137.7 to 155.6). There was strong evidence (*p* = 0.001) that the overall construction site injury rate per 100,000 workers of females was about one half the rate of males [rate ratio 0.56 (95%CI: 0.40 to 0.78)]. The fatal construction site injury rate per 100,000 workers of females was 37.8 (95%CI: 22.02 to 60.52) The fatal construction site injury rate per 100,000 workers of males was 33.15 (95%CI: 29.05 to 37.66). There was no evidence (*p* = 0.601) that the rates of fatal injuries differed in males and females (rate ratio 1.14 (95%CI: 0.70 to 1.87).

## Discussion

### Principal findings

We estimated that 17 female construction site workers sustained fatal injuries in Delhi in 2017. There was no evidence that fatal injury rates differed in males and females, however the overall construction site injury rate of females was over half that of males.

There is evidence for differences in the percentage ascertainment by FIRs of both fatal and non-fatal injuries according to gender: Overall, FIRs ascertained a greater proportion of injuries to female workers than to male workers. There was 100% ascertainment of non-fatal injuries to female workers by FIRs.

### Strengths and weaknesses of the study

Epidemiological evidence serves as a foundation for the planning of safety interventions, however, until this study, insufficient evidence on construction injuries in India was available. A strength of this study is that it included all accident cases reported to the Delhi police, ESIC and CWC from 1st January to 31st December 2017. Information bias was possible on account of non-availability of some FIRs on the website of the Delhi police, however, this was reduced by obtaining such FIRs directly from the police station concerned. Similarly, in the case of ESIC and CWC, efforts were made to obtain details of all the cases by visiting the offices personally. To the best of our knowledge, this is the first study to ascertain the burden of injuries on female construction workers in India. As construction injuries tend to be under reported, the capture-recapture method may help to estimate their true magnitude in a population. Since construction safety is a less researched area in India, this study bridges an important gap in the literature.

The capture-recapture method used in this study is based on a set of assumptions, and hence the failure to satisfy one or more of these assumptions may have led to incorrect estimates of the number of women injured in construction site accidents [21, 25]. The first assumption is that the study population should be closed. In our case, the capture and recapture samples took place at the same time (i.e. 2017) reducing chances of any change in the population between the two captures and thus the first assumption holds. Although it is possible that injured construction workers may cross the border of Delhi to get treatment in health facilities in the adjoining states, these injuries will still be included in the police records of Delhi as the incidents are registered in the police station having jurisdiction over the incident location. A second assumption is that both data sources should cover the same geographical area and time period. This assumption has been fully met as our study covers the whole of Delhi and the data were obtained from different sources for the same period. A third assumption is that the two sources of ascertainment should be independent and that members of the population have the same probability of being captured. In our case, the Delhi police, ESIC and CWC are independent and do not share data with each other. People report injuries to these organisations independently of each other, thus, this assumption is also met. A further assumption is the perfect identification of subjects of interest. This is fulfilled to a large extent as the police tend to record details of the injured accurately due to legal requirements. Similarly, people filing compensation claims with the Labour department and employers filing incident reports in the ESIC portal are also assumed to provide correct details; A further assumption is the perfect identification of common records without missed cases or false matches, i.e., perfect linkage of data from the two data sources: To fulfil this assumption, we took all possible care to ensure perfect linkage of records. A final assumption is homogeneity of capture; This means that all injuries should have the same probability of becoming known to the police as to the ESIC and the CWC. This assumption is also met as the employers are mandated by Law to report injuries to the police, ESIC and the CWC. If employers were under-reporting injuries sustained by their workers, it is likely that the probability of under-reporting injuries would not differ between these organisations. Although all efforts were made to fulfil these assumptions, there could still be certain weaknesses preventing assumptions to hold true.

The classification bias caused by inclusion of motor vehicle related accidents at construction sites by the police as road traffic accidents instead of construction site accidents introduces one more weakness. Another limitation of the study lies in the inherent limitation of police records as a source of information on injuries, including under-reporting, bias towards fatal and severe injuries, and recording of information without going into veracity of claims [26]. Another possible weaknesses of our study is the relatively short study period of 1 year.

### Strengths and weaknesses in relation to other studies

Our estimate of 258 fatal injuries in a year in Delhi was close to an earlier estimate of 256 fatal accidents every year at construction sites in Delhi between 2008 and 2012 [7]. We did not find any other study of injuries to female construction workers in India with which to compare our results. Our estimates of construction site injury incidence rates in Delhi- 143 injuries per 100,000 construction workers and 34 fatal injuries per 100,000 workers are, however, lower than estimates found in some high-income countries. For example, a study of work health and safety and workers’ compensation schemes in Australia and New Zealand found the incidence rate of serious claims in Australia was 16.0 per 1000 employees in 2015–16 [27]. In this study, however, the reported incidence included musculoskeletal disorders and diseases, in addition to occupational injuries [27]. Injury under-reporting has been found to be pervasive in the construction industry, especially in small establishments [28]. Our lower estimate of injury incidence in our study could thus be due to under-reporting of injuries to the police, the Labour Department, and the ESIC. Reasons for under-reporting found in other studies of construction site injuries include fear of negative consequences of reporting injuries, and workers’ perceptions of injuries as “part of the job.” [29] Although we did not test for homogeneity in capture, the differences we have observed in the reporting of injuries to the police and to ESIC/CWC suggests heterogeneity of capture which may have led to an underestimation of the total number of injuries in the population [30].

The capture recapture method has also highlighted the degree of underreporting of injuries to various authorities in Delhi. In case of police, the degree of underreporting estimated in this study was 57% for fatal, 70% for non-fatal and 64% for all injuries. In case of the combined data of ESIC and Commissioners of Workmen Compensation, the degree of under reporting of construction site injuries was even higher. It was 81.4% for fatal, 96.3% for non-fatal and 92% for all injuries.

Previous studies on completeness of ascertainment of unintentional injuries by police records in Lower-Middle-Income-Countries have found that police records ascertained between 4.2 to 77.8% of fatal injuries and 6.7 to 24.7% of non-fatal injuries [15, 31–39]. Ascertainment of non-fatal injuries in our study (30%) was higher, while ascertainment of fatal injuries (43%) is comparable with these studies. However, these previous studies have largely focused on road traffic injuries and none reported completeness of ascertainment of construction site injuries by police records. Our estimate of 258 fatal construction site injuries in a year in Delhi is also close to an earlier estimate of 256 fatal accidents every year at construction sites in Delhi between 2008 and 2012 [7].

### Meaning of the study: possible mechanisms and implications for policymakers

This study is the first to estimate the incidence of injuries to female construction site workers in India. Although rates of fatal injuries were the same in male and female construction workers, the overall injury rate of female construction workers was over half as great as the rate of males. This implies that female construction workers face a not insignificant risk whilst working alongside their male counterparts and hence safety measures (e.g., personal protective equipment) that are appropriate and culturally acceptable to Indian women are needed. However, given the susceptibility of women workers to be laid off or under-employed, it is possible that training and safety equipment requirements might provide a further obstacle to their employment within construction.

It is possible that some female workers are at increased injury risk as they are required to attend to their children playing at the construction site [1]. The Building and Other Construction Workers (Regulation of Employment and Conditions of Service) Act 1996, provides for setting up of childcare facilities at construction sites where more than 50 female workers are ordinarily employed [16]. There is possibly a need for stricter enforcement of this provision. The legislation might also be amended to make provision of childcare facilities at construction sites where ordinarily more than 5 mothers are employed. Another possible reason behind injuries sustained by female construction workers could be a lack of education and training of female workers [40]. If so, this may underscore the need for enhanced focus on training of female construction workers.

The differences in ascertainment by FIRs of fatal and non-fatal injuries according to gender could be due to under-reporting to the ESIC and the CWC of non-fatal injuries sustained by female construction workers which could have biased our estimate of the total numbers of injuries: In capture-recapture analysis, if one of the sources of data captures very few cases, the analysis is likely to produce a biased estimate of the population size [41]. Since the number of non-fatal injuries reported by female construction workers to the ESIC and the CWC was zero, the capture-recapture method may have under-estimated the number of non-fatal injuries suffered by female construction workers, and consequently resulted in a higher percentage of their ascertainment by the FIRs.

The study also points towards substantial underreporting of injuries to various authorities despite the legal mandate. The underreporting was higher for the Employee State Insurance Corporation and Commissioners of Workmen Compensation. This implies that the construction workers are being denied the entitled compensation, and insurance benefits. To prevent information from reaching enforcement agencies, the employers also try to get the workers treated at construction sites, compromising on the quality of care, which may have serious health consequences. It also informs that the employers are not being punished for various acts of their negligence which are leading to accidents and injuries at the construction sites. The results of this study also point towards an immediate need to create awareness about the legal provisions regarding reporting of injuries and stricter measures to enforce the laws. The construction industry in India is under-supervised due to country wide shortage of Labour Inspectors and other enforcement staff.

The study also underscores the need to have a comprehensive injury prevention policy in India. It highlights the need for creation of a unified authority for injury prevention and mitigation under the Ministry of Health and Family Welfare in India, a responsibility scattered across multiple departments.

### Unanswered questions and future research

Our research was confined to Delhi, the capital of India. For a national picture of the burden of injuries to female construction workers, a national study is needed. Findings of such a study may stimulate appropriate policy responses at the national level as well as in different states of India. Estimates of the injury burden and gender differences may be validated in future studies using other sources of data on injuries for the ‘recapture’ sample (e.g., hospital records, ambulance records, population surveys etc.). Future research might also focus on the burden of other occupational injuries (e.g., industrial workers, hospital staff, agricultural workers etc.) using a similar research design.

## Data Availability

The datasets used and analysed during the current study are available from the corresponding author on reasonable request.
